# Transcranial Doppler After Successful Endovascular Revascularization and Hospitalization Outcomes

**DOI:** 10.1161/SVIN.122.000785

**Published:** 2023-05-24

**Authors:** Victor J. Del Brutto, Jacob A. Sambursky, Nastajjia A. Krementz, Faisal J. Gondal, Hannah E. Gardener, Frank Cabrera, Yosdely Cabrera, Faddi Saleh Velez, Jose G. Romano, Sebastian Koch

**Affiliations:** ^1^ Department of Neurology University of Miami Miller School of Medicine Miami FL; ^2^ Quality and Patient Safety Department Jackson Health System Miami FL; ^3^ Department of Clinical Neurophysiology Jackson Health System Miami FL

**Keywords:** acute ischemic stroke, large‐vessel occlusion, transcranial Doppler

## Abstract

**Background:**

Almost half of large‐vessel occlusion strokes have unfavorable outcomes despite successful endovascular therapy. We aim to investigate whether postrevascularization cerebral hemodynamics, determined by transcranial Doppler (TCD), associate with hospitalization outcomes in this population.

**Methods:**

The current observational cohort study analyzed 155 patients with successfully revascularized anterior circulation large‐vessel occlusion stroke (mean age, 68.3±15.4 years; 55% women) who had TCD within 48 hours from endovascular therapy. TCD parameters (mean flow velocity, peak systolic velocity, and pulsatility index) were recorded at the ipsilateral middle cerebral artery, and blood flow signals were categorized using the Thrombolysis in Brain Ischemia grades into normal (grade 5), stenotic (grade 4), or dampened (grade ≤3). Hospitalization outcomes comprised favorable discharge modified Rankin Scale score (0–2), favorable discharge destination (home or acute inpatient rehabilitation), and in‐hospital mortality. Logistic regression models adjusted for age, initial National Institutes of Health Stroke Scale score, and Alberta Stroke Program Early CT [Computed Tomography] Score were fit to determine TCD findings in association with study outcomes.

**Results:**

Abnormal TCD‐derived blood flow was found in 54 (35%) cases, including 35 (23%) with Thrombolysis in Brain Ischemia grade 4 and 19 (12%) with Thrombolysis in Brain Ischemia grade ≤3. Overall, 31% had favorable discharge modified Rankin Scale score, 65% had favorable destination, and 14% died. Thrombolysis in Brain Ischemia grade ≤3 was associated with lower likelihood of both favorable discharge modified Rankin Scale score (adjusted odds ratio [OR], 0.09 [95% CI, 0.01–0.81]) and favorable destination (adjusted OR, 0.22 [95% CI, 0.07–0.71]). Mean flow velocity and peak systolic velocity were not associated with study outcomes. Conversely, increased pulsatility index was inversely associated with favorable destination (adjusted OR, 0.34 [95% CI, 0.13–0.87]).

**Conclusions:**

TCD after successful endovascular therapy identified abnormal blood flow in one‐third of cases. Dampened flow and markers of increased microvascular resistance were associated with unfavorable hospitalization outcomes. TCD could provide valuable prognostic information in this population and identify potential therapeutic targets.

## Graphical abstract

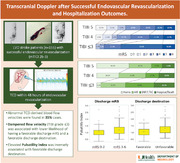

**Nonstandard Abbreviations and Acronyms**
ASPECTS: Alberta Stroke Program Early CT [Computed Topography] Score
EVT: endovascular therapy
ICH: intracranial hemorrhage
LVO: large‐vessel occlusion
MCA: middle cerebral artery
MFV: mean flow velocity
mRS: modified Rankin Scale
mTICI: modified Thrombolysis in Cerebral Infarction
NIHSS: National Institutes of Health Stroke Scale
PI: pulsatility Index
TCD: transcranial Doppler
TIBI: Thrombolysis in Brain Ischemia

**Clinical Perspective**

**What Is New?**

- Transcranial Doppler (TCD) within 48 hours after successful large‐vessel occlusion endovascular revascularization identified abnormal blood flow velocities in nearly one‐third of cases.
- In patients with large‐vessel occlusion stroke, post–endovascular therapy TCD‐derived dampened flow velocities and markers of increased microvascular resistance (ie, elevated pulsatility index) are associated with worse hospitalization outcomes despite successful revascularization, independent of patient's age, presenting stroke severity, or infarct burden on initial computed tomography scan.

**What Are the Clinical Implications?**

- TCD performed early after endovascular therapy has potential value for prognostication.
- TCD provides real‐time evidence of the pathological mechanisms (ie, increased microvascular resistance) associated with poor outcomes in patients with large‐vessel occlusion stroke despite successful macrovascular revascularization.
- Optimization of TCD‐derived markers associated with unfavorable outcomes could serve as therapeutic targets to improve medical management after endovascular revascularization.

Large‐vessel occlusion (LVO) is found in 24% to 38% of all acute ischemic strokes and carries a higher risk of death and functional dependence when compared with non‐LVO ischemic strokes.^1^, ^2^ Pivotal randomized controlled trials have established endovascular therapy (EVT) efficacy in selected patients with LVO stroke up to 24 hours from symptoms onset.^3^, ^4^, ^5^ Despite EVT demonstrated benefit, near half of EVT‐eligible patients with LVO who reach successful revascularization (modified Thrombolysis in Cerebral Infarction [mTICI] score of 2b–3) continue to be disabled or die at 3 months.^6^, ^7^ The lack of favorable outcomes in this population remains poorly understood. Nonmodifiable factors, such as premorbid brain reserve and the size and location of the infarct core before EVT, are known to be associated with poor outcomes.^8^ Impaired cerebral hemodynamics after EVT may also contribute to unfavorable outcomes. Both decreased and increased cerebral blood flow may lead to penumbra hypoperfusion or reperfusion injury, respectively.^9^ Moreover, impaired microvascular tissue‐level reperfusion despite upstream macrovascular revascularization, known as the no‐reflow phenomenon, has been proposed as a common and clinically relevant pathological process in LVO strokes after EVT.^10^

Transcranial Doppler (TCD) is a noninvasive and readily available bedside tool able to measure blood flow velocities as cerebral blood flow surrogate and aid in the understanding of cerebral hemodynamics after EVT.^11^ There is growing evidence on the association between TCD‐derived abnormal blood flow velocities after EVT and increased risk of post‐EVT intracranial hemorrhage (ICH),^12^, ^13^ larger infarct volume,^14^ brain edema severity,^15^ and poor 90‐day outcomes.^12^, ^16^ In addition, the Gosling pulsatility index (PI), derived from TCD, is an established marker of microvascular resistance that could help identify no‐reflow phenomenon early after EVT.^17^, ^18^ Therefore, our study aims to examine the value of TCD in the evaluation of cerebral hemodynamics early after successful endovascular revascularization and investigate the association between TCD parameters and hospitalization outcomes in patients with anterior circulation LVO stroke.

## Methods

The data that support the findings of this study are available from the corresponding author on reasonable request.

### Study Population

We prospectively included consecutive patients with acute ischemic stroke with LVO of the anterior circulation who underwent successful endovascular revascularization at a single large comprehensive stroke center between January 2021 and June 2022. During this period, TCD was routinely obtained within 48 hours of EVT per standard hospital practices. In addition, we retrospectively reviewed our electronic database between January 2010 and December 2020 to identify patients with LVO who had TCD completed on the basis of clinical judgment within 48 hours of EVT. Study inclusion criteria were as follows: (1) acute stroke secondary to LVO of the intracranial internal carotid artery, middle cerebral artery (MCA) M1 segment, or MCA proximal M2 segment, (2) EVT within 24 hours of symptom onset achieving successful revascularization defined by mTICI score of 2b to 3, and (3) TCD evaluation within 48 hours after EVT. For this analysis, we excluded patients aged <18 years and those without adequate acoustic temporal windows. The Institutional Review Board of the University of Miami approved the protocol, and waiver of informed consent was accepted. This study followed the Strengthening the Reporting of Observational Studies in Epidemiology reporting guidelines for observational studies.

Clinical data were collected on the basis of medical records review and comprised demographics (age at presentation, sex, race, and ethnicity), history of vascular risk factors (hypertension, diabetes, hyperlipidemia, and current smoking), initial stroke severity, as determined by the National Institutes of Health Stroke Scale (NIHSS), presenting infarct burden assessed on the initial computed tomography scan using the Alberta Stroke Program Early CT [Computed Topography] Score (ASPECTS), and stroke cause diagnosis documented on the discharge summary note. EVT procedure‐related characteristics collected included intravenous thrombolysis bridging therapy, time to puncture in minutes, number of passes, and the degree of final angiographic reperfusion, as determined by the mTICI score.^19^

### TCD Examination

TCD studies were performed by 1 of 2 certified ultrasound technologists (F.C. or Y.C.) using a 2‐MHz pulsed Doppler ultrasonography instrument (Image Monitoring USA Inc Dolphin IQ). TCD findings were interpreted by a neurosonology‐certified vascular neurologist blinded to medical information. Our institutional protocol included standard bilateral isonation through the temporal acoustic window of the anterior, posterior, and middle cerebral arteries, as well as terminal internal carotid artery throughout their course at 2‐mm intervals. For the purposes of this study, we recorded the mean flow velocity (MFV), peak systolic velocity, and the PI at the ipsilateral (recanalized) MCA at the exact or nearest depth of 54 mm corresponding to the distal MCA M1 segment. Ipsilateral MCA residual flow signals were graded according to the Thrombolysis in Brain Ischemia (TIBI) classification into absent flow (grade 0), minimal systolic spike without diastolic flow (grade 1), blunted or flattened systolic flow acceleration compared with the contralateral side (grade 2), normal systolic flow acceleration and positive end‐diastolic velocity but with MFV <30% compared with the contralateral side (grade 3), stenotic flow defined by MFV >80 cm/s and >30% compared with the contralateral side (grade 4), and normal flow when the waveform was similar and MFV <30% divergent from the contralateral side (grade 5).^20^ For analysis, TIBI grades were categorized as dampened (grade ≤3), stenotic (grade 4), or normal (grade 5).

### Hospitalization Outcomes

Primary hospitalization outcomes comprised the proportion of patients with favorable functional status at discharge, favorable discharge destination, and in‐hospital mortality. Functional status was assessed by the discharge modified Rankin Scale (mRS) score and dichotomized as favorable (mRS score, 0–2) or unfavorable (mRS score, 3–6). A favorable destination was defined by being discharged home or to acute inpatient rehabilitation, whereas unfavorable destination included skilled nursing facility, long‐term acute care facility, hospice, or death. Discharge destination is a strong predictor of 90‐day functional outcome in patients with stroke,^21^, ^22^, ^23^ with 1 study showing that the positive likelihood of 90‐day mRS score of 3 to 6 after skilled nursing facility destination was 13‐ and 9‐fold higher when compared with home and acute inpatient rehabilitation, respectively.^21^ Additional study outcomes included early neurological improvement (ENI), defined by a reduction of ≥6 points or a total of 0 to 2 points on the 24‐hour post‐EVT NIHSS, as well as post‐EVT symptomatic ICH, defined by the presence of ICH associated with NIHSS deterioration of ≥4 points.

### Statistical Analysis

Data analysis was performed using SAS v9.4 (SAS Institute, Cary, NC). Descriptive statistics are presented as means with SDs or medians with interquartile ranges for continuous variables, and as percentages for categorical variables. In univariate analyses, continuous variables were compared by linear models, and categorical variables were compared by χ^2^ or the Fisher exact test, as appropriate. Patients with missing information in any variable were excluded from individual analyses. We investigated the hypothesis that TCD parameters (MFV, peak systolic velocity, and PI) as well as abnormal TIBI grades (TIBI ≤3 and TIBI 4) are associated with hospitalization outcomes, holding constant patient's age, initial stroke severity, and presenting infarct burden. For this purpose, we fit binary logistic regression models, adjusted for age, initial NIHSS score, and ASPECTS, with TCD findings as the independent variable and hospitalization outcomes as the dependent variable to yield adjusted odd ratios (aORs) with 95% CIs. Complementary logistic regression models further adjusted for mTICI score and site of vessel occlusion to test whether results remained unchanged. Differences were considered statistically significant at *P*<0.05.

## Results

Between January 2021 and June 2022, 72 of 124 consecutive patients with anterior circulation LVO stroke who underwent EVT at our center fulfilled study criteria. Reasons for exclusion included incomplete revascularization in 13, inadequate temporal windows or incomplete TCD data in 24, and TCD not performed within 48 hours after EVT in 15. Retrospective review of medical records from January 2010 to December 2020 identified 110 patients with LVO stroke treated with EVT who had TCD within 48 hours. Of these, 27 patients were excluded (20 with inadequate temporal bone window and 7 with incomplete recanalization), leaving 83 additional patients for study inclusion. The total study sample comprised 155 patients (mean age, 68.3±15.4 years; 55% women). The median time to TCD was 19.1 hours (interquartile range, 17.2 hours). Baseline characteristics of the study population are summarized in Table 1. When compared with the prospective cohort, patients in the retrospective series were less likely to be men, non‐Hispanic Black race and ethnicity, and nonsmokers. There was a trend toward higher frequency of normal TCD‐derived flow velocities (TIBI grade 5) in the prospective cohort, but there were no significant differences in clinical presentation or EVT procedure‐related characteristics (Supplemental Table S1).

**Table 1:** Baseline Characteristics of Patients With LVO Acute Ischemic Stroke With Successful Endovascular Revascularization Included in This Study

Variable	Value (n=155)	
Demographics		
Age median (SD), y	68	(15)
Women, n (%)	85	(55)
Race and ethnicity, n (%)		
Non‐Hispanic White	44	(28)
Non‐Hispanic Black	17	(11)
Hispanic	80	(51)
Other*	14	(9)
Vascular risk factors, n (%)		
Hypertension	117	(76)
Diabetes	52	(34)
Hyperlipidemia	58	(38)
Current smoker	35	(23)
Previous stroke	26	(17)
Clinical presentation		
Premorbid mRS score, n (%)		
0	109	(70)
1	28	(18)
2	18	(12)
NIHSS score, median (IQR)	15	(10)
Occluded vessel, n (%)		
ICA	27	(17)
M1	84	(54)
M2	44	(28)
ASPECTS, median (IQR)	9	(2)
Stroke cause, n (%)		
Cardioembolic	65	(42)
Large‐artery disease	22	(14)
Undetermined	59	(38)
Other*	9	(6)
Endovascular therapy		
IV‐tPA bridging therapy, n (%)	72	(47)
Time to puncture, median (IQR), min	94	(55)
First‐pass revascularization, n (%)	83	(54)
mTICI score, n (%)		
2b	37	(24)
2c	29	(19)
3	89	(57)
Transcranial Doppler		
TIBI grades, n (%)		
≤3	19	(12)
4	35	(23)
5	101	(65)
MFV, mean (SD), cm/s	61	(32)
PSV, mean (SD), cm/s	105	(47)
PI, mean (SD)	1.2	(0.4)

ASPECTS indicates Alberta Stroke Program Early CT [Computed Tomography] Score; ICA, internal carotid artery; IQR, interquartile range; IV‐tPA, intravenous tissue‐type plasminogen activator; LVO, large‐vessel occlusion; M1, middle cerebral artery M1 segment; M2, middle cerebral artery M2 segment; MFV, mean flow velocity; mRS, modified Rankin Scale; mTICI, modified Thrombolysis in Cerebral Infarction; NIHSS, National Institutes of Health Stroke Scale; PI, pulsatility index; PSV, peak systolic velocity; and TIBI, Thrombolysis in Brain Ischemia.

*Other race include American Indian or Alaska Native, Asian, Native Hawaiian or Pacific Islander.

The median length of hospitalization was 9 days (interquartile range, 11 days). Forty‐eight (31%) patients had favorable discharge mRS score, 100 (65%) had favorable destination, and 22 (14%) died during hospitalization. In univariate analyses, both favorable discharge mRS score and favorable destination were associated with younger age, lower initial NIHSS score, lower frequency of cardioembolic stroke cause, and higher rate of first‐pass revascularization. Presenting ASPECTS was significantly higher among those with favorable discharge mRS score and showed a trend toward higher values among those with favorable destination. In‐hospital mortality was associated with higher initial NIHSS score and higher frequency of cardioembolic stroke cause (Table 2).

**Table 2:** Baseline Characteristics, Clinical Presentation, and TCD Characteristics According to Hospitalization Outcomes

Variable	Discharge mRS score			Discharge destination			In‐hospital mortality		
	0–2 (n=48)	3–6 (n=107)	*P* value	Favorable (n=100)	Unfavorable (n=55)	*P* value	Yes (n=22)	No (n=133)	*P* value
Demographics									
Age, median (SD), y	63.3 (14.9)	70.5 (15.2)	0.01	65.5 (15.4)	73.3 (14.3)	0.00	71.6 (14.6)	67.7 (15.6)	0.28
Women, n (%)	17 (35.1)	53 (49.5)	0.10	46 (46.0)	24 (43.6)	0.78	7 (31.8)	63 (47.4)	0.07
Race and ethnicity, n (%)									
Non‐Hispanic White	19 (39.6)	25 (23.4)	0.17	35 (35.00)	9 (16.36)	0.08	2 (9.09)	42 (31.58)	0.10
Non‐Hispanic Black	3 (6.3)	14 (13.1)		11 (11.00)	6 (10.91)		3 (13.64)	14 (10.53)	
Hispanic	22 (45.8)	58 (54.2)		45 (45.00)	35 (63.64)		13 (59.09)	67 (50.38)	
Other*	4 (8.3)	10 (9.4)		9 (9.00)	5 (9.09)		4 (18.18)	10 (7.52)	
Vascular risk factors, n (%)									
Hypertension	36 (75.0)	81 (75.7)	0.93	74 (74.0)	43 (78.2)	0.56	17 (77.3)	100 (75.2)	0.83
Diabetes	16 (33.3)	36 (33.6)	0.97	30 (30.0)	22 (40.0)	0.21	9 (40.9)	43 (32.3)	0.43
Hyperlipidemia	21 (43.8)	37 (34.6)	0.28	37 (37.0)	21 (38.2)	0.88	6 (27.3)	52 (39.1)	0.29
Current smoker	13 (27.1)	22 (20.6)	0.37	26 (26.0)	9 (16.4)	0.17	6 (27.3)	29 (21.8)	0.59
Previous stroke	6 (12.5)	20 (18.7)	0.34	14 (14.0)	12 (21.8)	0.21	5 (22.4)	21 (15.8)	0.54
Clinical presentation									
NIHSS score, median (IQR)	10.5 (7.5)	16 (8)	0.00	14 (8)	19 (9)	0.00	20.5 (8)	14 (9)	0.00
Occluded vessel, n (%)									
ICA	6 (12.5)	21 (19.6)	0.20	17 (17.0)	10 (18.2)	0.22	4 (18.2)	23 (17.3)	0.50
M1	24 (50.0)	60 (56.1)		50 (50.0)	34 (61.8)		14 (63.6)	70 (52.6)	
M2	18 (37.5)	26 (24.3)		33 (33.0)	11 (20.0)		4 (18.2)	40 (30.1)	
ASPECTS, median (IQR)	9 (2)	9 (1)	0.01	9 (2)	9 (1)	0.07	8.5 (1)	9.0 (2)	0.37
Stroke cause, n (%)									
Cardioembolic	16 (33.3)	49 (45.8)	0.04	34 (34.0)	31 (56.4)	0.03	15 (68.2)	50 (37.6)	0.02
Large‐artery disease	8 (16.7)	14 (13.1)		15 (15.0)	7 (12.7)		2 (9.1)	20 (15.0)	
Undetermined	24 (50.0)	35 (32.7)		46 (46.0)	13 (23.6)		3 (13.6)	56 (42.1)	
Other	0 (0.0)	9 (8.4)		5 (5.0)	4 (7.3)		2 (9.1)	7 (5.3)	
Endovascular therapy									
IV‐tPA bridging therapy, n (%)	23 (47.9)	49 (45.79)	0.81	51 (51.0)	21 (38.2)	0.13	10 (45.45)	62 (46.62)	0.92
Time to puncture, median (IQR), min	108 (101)	87.5 (46)	0.09	97 (57)	90 (47)	0.32	90 (75)	95 (55)	0.79
First‐pass revascularization, n (%)	31 (66.0)	52 (49.1)	0.03	58 (59.2)	25 (45.5)	0.05	9 (40.9)	74 (56.5)	0.17
mTICI score, n (%)									
2b	10 (20.8)	27 (25.2)	0.25	20 (20.0)	17 (30.9)	0.30	4 (18.2)	33 (24.8)	0.75
2c	6 (12.5)	23 (21.5)		19 (19.0)	10 (18.2)		5 (22.7)	24 (18.1)	
3	32 (66.7)	57 (53.3)		61 (61.0)	28 (50.9)		13 (59.1)	76 (57.1)	
TCD findings									
TIBI grades, n (%)									
≤3	1 (2.1)	18 (16.8)	0.02	7 (7.0)	12 (21.2)	0.02	3 (13.6)	16 (12.0)	0.98
4	9 (18.8)	26 (24.3)		22 (22.0)	13 (23.6)		5 (22.7)	30 (22.6)	
5	38 (79.2)	63 (58.9)		71 (71.0)	30 (54.6)		14 (63.6)	87 (65.4)	
MFV, mean (SD), cm/s	66.3 (39.3)	59.0 (27.6)	0.18	63.4 (31.9)	57.26 (31.32)	0.25	63.7 (30.5)	60.8 (32.0)	0.69
PSV, mean (SD), cm/s	108.6 (50.6)	104.0 (45.7)	0.58	105.3 (43.6)	105.7 (53.4)	0.95	118.2 (50.2)	103.3 (46.5)	0.17
PI, mean (SD)	1.1 (0.4)	1.2 (0.5)	0.08	1.1 (0.3)	1.3 (0.5)	0.00	1.3 (0.4)	1.1 (0.4)	0.07

ASPECTS indicates Alberta Stroke Program Early CT [Computed Tomography] Score; ICA, internal carotid artery; IQR, interquartile range; IV‐tPA, intravenous tissue‐type plasminogen activator; M1, middle cerebral artery M1 segment; M2, middle cerebral artery M2 segment; MFV, mean flow velocity; mRS, modified Rankin Scale; mTICI, modified Thrombolysis in Cerebral Infarction; NIHSS, National Institutes of Health Stroke Scale; PI, pulsatility index; PSV, peak systolic velocity; TCD, transcranial Doppler; and TIBI, Thrombolysis in Brain Ischemia.

*Other race include American Indian or Alaska Native, Asian, Native Hawaiian or Pacific Islander.

**Figure 1:**
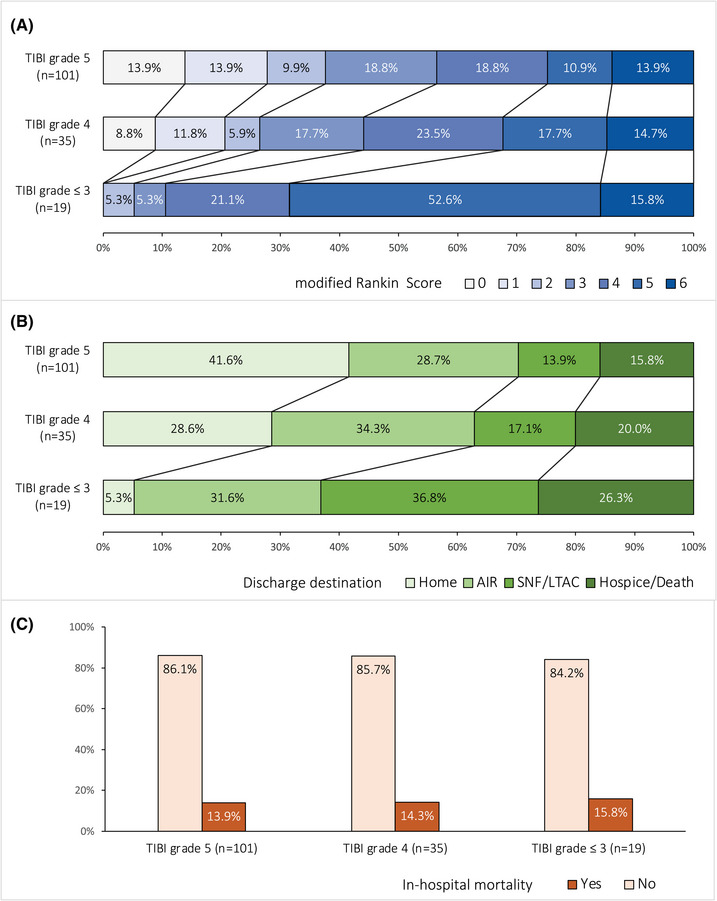
**Hospitalization outcomes according to 48‐hour transcranial Doppler‐derived Thrombolysis in Brain Ischemia (TIBI) grades**. **A**, Modified Rankin Scale score at discharge. **B**, Discharge destination. **C**, In‐hospital mortality. AIR indicates acute inpatient rehabilitation; LTAC, long‐term acute care; and SNF, skilled nursing facility.

**Figure 2:**
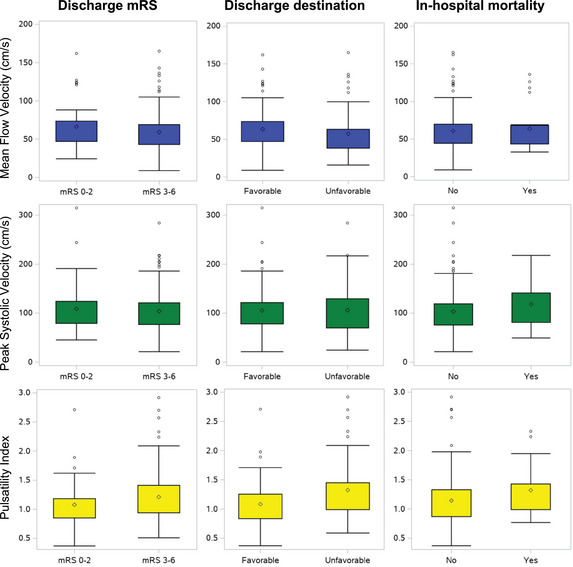
**Distribution of 48‐hour transcranial Doppler parameters at the ipsilateral middle cerebral artery according to the modified Rankin Scale (mRS) score at discharge, discharge destination, and in‐hospital mortality**.

TCD analysis revealed that 54 (35%) patients had abnormal TIBI grades despite successful revascularization, including 35 (23%) with stenotic flow (TIBI grade 4) and 19 (12%) with dampened flow (TIBI grade ≤3). The frequency of favorable discharge mRS score and favorable destination was lower in those with abnormal TIBI grades, but there were no differences in in‐hospital mortality (Figure 1). After adjusting for age, initial NIHSS score, and presenting ASPECTS, TIBI grade ≤3 remained associated with lower likelihood of both favorable discharge mRS score (aOR, 0.09 [95% CI, 0.01–0.81]) and favorable destination (aOR, 0.22 [95% CI, 0.07–0.71]; Table 3). There were no significant differences in MFV and peak systolic velocity mean values with regard to hospitalization outcomes. However, PI mean values were consistently higher among those with unfavorable discharge mRS score, unfavorable discharge destination, and in‐hospital mortality (Figure 2). In adjusted analyses, increased PI values remained inversely associated with favorable destination (aOR, 0.34 [95% CI, 0.13–0.87]; Table 3). Complementary analyses that further adjusted for mTICI score and site of vessel occlusion did not alter study results (Supplemental Table S3).

**Table 3:** In‐Hospital Outcomes Based on TCD Parameters and TIBI Flow Grades Measured Within 48 Hours of EVT

Variable	Discharge mRS score of 0–2	Favorable destination	In‐hospital mortality	Early neurological improvement	Symptomatic ICH
TIBI grades					
TIBI grade 4 vs 5	0.69 (0.26–1.85)	0.89 (0.36–2.21)	0.74 (0.29–2.40)	0.61 (0.27–1.38)	1.25 (0.33–4.7)
TIBI grade ≤3 vs 5	0.09 (0.01–0.81)*	0.22 (0.07–0.71)*	0.87 (0.21–3.64)	0.19 (0.05–0.71)*	2.91 (0.73–11.66)
TCD parameters					
MFV, cm/s	1.01 (0.99–1.02)	1.01 (0.99–1.02)	1.00 (0.99–1.02)	1.01 (0.99–1.02)	1.01 (0.97–1.01)
PSV, cm/s	1.00 (0.99–1.01)	1.00 (0.99–1.01)	1.01 (0.97–1.04)	1.00 (0.99–1.01)	1.00 (0.98–1.01)
PI	0.68 (0.23–2.06)	0.34 (0.13–0.87)*	2.00 (0.73–5.43)	0.47 (0.19–1.16)	2.26 (0.78–6.60)

Data are given as adjusted odds ratio (95% CI). Logistic regression model adjusted for age at presentation, initial National Institutes of Health Stroke Scale score, and Alberta Stroke Program Early CT [Computed Tomography] Score; adjusted odds ratios for MFV, PSV, and PI are per 1‐unit increase. EVT indicates endovascular therapy; ICH, intracranial hemorrhage; MFV, mean flow velocity; mRS, modified Rankin Scale; PI, pulsatility index; PSV, peak systolic velocity; TCD, transcranial Doppler; and TIBI, Thrombolysis in Brain Ischemia.

^*^ *P*<0.05.

ENI and post‐EVT symptomatic ICH were seen in 66 (43%) and 16 (10%) of cases, respectively. ENI was associated with higher presenting ASPECTS, greater frequency of first‐pass revascularization, and having normal residual flow velocities on TCD (TIBI grade 5), whereas post‐EVT symptomatic ICH was associated with lower presenting ASPECTS and elevated PI values (Supplemental Table S2). TIBI grade ≤3 remained associated with a lower likelihood of ENI in adjusted analysis (aOR, 0.19 [95% CI, 0.05–0.71]; Table 3).

## Discussion

In our study, TCD performed early after successful endovascular revascularization identified abnormal MCA residual blood flow velocities in nearly one‐third of patients with stroke with anterior circulation LVO. Having dampened flow velocities (TIBI grade ≤3) was associated with lower likelihood of being functionally dependent at discharge or having a favorable discharge destination independent of age, initial stroke severity, and presenting infarct burden. Moreover, increased markers of microvascular resistance were higher among those with unfavorable hospitalization outcomes, thus suggesting microvasculature no reflow may contribute to poor outcomes despite good macrovascular revascularization.

Similar to our results, 1 study across 193 successfully revascularized (TICI grade 2b–3) patients with LVO stroke demonstrated that TCD performed within 72 hours of revascularization displayed abnormal residual MCA blood flow velocities (TIBI 0–4) in 36% of cases, and abnormal TIBI grades were an independent predictor of worse 90‐day outcomes.^16^ Compared with the aforementioned study, our results identified dampened blood flow velocities (TIBI grade ≤3) as a TCD‐derived biomarker strongly associated with unfavorable outcomes . Aligned with our observation, 1 recent study found that post‐EVT decreased TCD velocities correlated with brain edema severity in patients with LVO stroke.^15^ Dampened blood flow velocities could be attributed to impending vessel reocclusion, systemic conditions predisposing to cerebral hypoperfusion (ie, arterial hypotension), increased distal resistance attributable to clot migration or no‐reflow phenomenon, large infarcted brain volumes resulting in reduced metabolic demand, or a combination of the above. On the other hand, post‐EVT increased TCD velocities have also been associated with hemorrhagic transformation, larger infarct volumes, and, ultimately, worse functional outcomes.^12^, ^13^, ^14^ These findings coincide with magnetic resonance imaging–based observational studies that showed an association between postreperfusion hyperperfusion and hemorrhagic transformation,^24^, ^25^ and suggest that TCD is a useful tool for detecting early signs of impaired cerebral autoregulation and reperfusion injury after EVT.^26^ In our study, the proportion of patients with abnormally increased MFV defined by TIBI grade 4 was numerically higher among those with unfavorable discharge mRS score, absent ENI, and post‐EVT symptomatic ICH. However, those differences did not reach statistical significance.

We found that PI values were consistently higher among those with unfavorable hospitalization outcomes. Consistent with our findings, 2 investigations across populations similar to ours found that post‐EVT elevated MCA PI values correlated with worse 90‐day functional outcomes.^17^, ^18^ As aforementioned, these findings may be attributed to the fact that PI is a marker of microvascular resistance, and higher values after successful EVT result from impaired microvascular perfusion attributable to obstruction from pericyte contraction, endothelial cell swelling, and luminal clogging with leukocytes and microthrombi (the no‐reflow phenomenon).^27^, ^28^, ^29^ Post‐EVT perfusion neuroimaging has shown that 25% of revascularized LVOs had radiological evidence of cerebral no reflow, and this associates with hemorrhagic transformation, greater infarct volume, and worse 90‐day outcomes.^10^ Further evidence on the clinical relevance of impaired microvasculature reperfusion despite macrovascular revascularization is provided by the CHOICE (Chemical Optimization of Cerebral Embolectomy) randomized clinical trial, which showed that adjunct intraarterial alteplase after successful endovascular revascularization resulted in a greater likelihood of excellent neurological outcomes at 90 days.^30^ CHOICE trial investigators suggested that the benefit of postrevascularization thrombolytics was likely explained by improvement in microcirculatory reperfusion.^30^

Our findings, along with the aforementioned published data, have several clinical implications. First, current evidence supports TCD potential value for prognostication after EVT. Second, TCD may be a practical tool for the early detection of post‐EVT complications that require emergent therapeutic interventions, such as vessel reocclusion, brain edema, or hemorrhagic transformation. Third, TCD can provide real‐time evidence of the pathological mechanisms associated with poor outcomes in patients with LVO despite successful revascularization. Finally, optimization of TCD‐derived biomarkers associated with unfavorable outcomes could be a potential therapeutic target to improve post‐EVT medical management. For the last point, patients with TCD parameter improvement between admission and follow‐up have had significant clinical recovery compared with patients whose TCD findings remained abnormal.^31^ Moreover, small controlled trials have shown that among patients with LVO who underwent EVT, TCD‐guided blood pressure management focused to normalization of MCA flow velocities was associated with better functional outcomes compared with guidelines‐based blood pressure management.^32^, ^33^ Emerging TCD techniques using robotically assisted devices and automated morphological characterization of TCD waveforms may overcome the logistic difficulties of continuous TCD monitoring in patients with stroke after EVT.^34^

Our study has several limitations. First, our sample size is relatively small, and nearly half of patients were identified retrospectively based on TCDs ordered at the physician's discretion. Therefore, sample bias cannot be excluded. Second, despite choosing a 48‐hour window after EVT, the greatest value of TCD is likely to be found in the hyperacute phase (immediately after EVT) when the potential benefit of therapeutic interventions is the highest. Third, our study exclusively included anterior circulation LVO strokes, thus restraining generalizability to LVO of the posterior circulation. Fourth, we used hospitalization outcomes because of their clinical relevance. However, we lacked 90‐day functional outcomes, as well as systematic neuroimaging follow‐up that could provide better insight on the association between TCD findings and final infarct volume. Finally, we present associational models that do not quantify the risk of unfavorable outcomes at the individual level. Future studies applying predictive modeling in larger data sets including additional post‐EVT variables, such as 24‐hour NIHSS score and imaging findings, are needed to better quantify the predictive value of TCD after successful EVT.

In summary, TCD evaluation early after EVT frequently identified abnormal residual flow velocities despite successful revascularization. TCD‐derived dampened flow velocities and increased markers of microvasculature resistance were particularly associated with unfavorable hospitalization outcomes. These TCD‐derived biomarkers may prove to be valuable for prognostication after EVT. Furthermore, TCD can help recognize pathological mechanisms responsible of poor outcomes despite timely revascularization and expose novel therapeutic targets. Continuous TCD monitoring initiated immediately after EVT is a promising approach to deliver TCD‐guided therapeutic interventions focused on normalizing residual flow velocities. Yet, more research is needed in this field.

## Sources of Funding

None.

## Disclosures

Victor J. Del Brutto, Jacob A. Sambursky, Nastajjia A. Krementz, Faisal J. Gondal, Hannah E. Gardener, Frank Cabrera, Yosdely Cabrera, Faddi Saleh Velez, Jose G. Romano, and Sebastian Koch have no relevant conflict of interest to disclose.

## Supporting information

Supplemental Tables S1, S2, and Table S3.
Supporting Information.
