# Supplementary material for: Transcranial Doppler After Successful Endovascular Revascularization and Hospitalization Outcomes
Source: Stroke Vasc Interv Neurol. 2023 May 24;3(4):e000785. doi: 10.1161/SVIN.122.000785 (PMC12778674; doi:10.1161/SVIN.122.000785)
Supplement: Supplementary file 1 — Supplemental Tables S1, S2, and Table S3. Supporting Information. [file SVI2-3-e000785-s001.pdf]

Supplemental Material

Transcranial Doppler after Successful Endovascular Revascularization and Hospitalization Outcomes.

Victor J. Del Brutto, MD, MS,[1] Jacob A. Sambursky, MD,[1] Nastajjia A. Krementz, MD,[1] Faisal J. Gondal, MBBS,[2] Hannah E. Gardener, ScD,[1] Frank Cabrera, MD,[3] Yosdely Cabrera, MD, RN,[3] Faddi Saleh Velez, MD,[1] Jose G. Romano, MD,[1] Sebastian Koch, MD.[1]

[1] Department of Neurology, University of Miami Miller School of Medicine, Miami, FL
[2] Quality and Patient Safety Department, Jackson Health System, Miami, FL
[3] Department of Clinical Neurophysiology, Jackson Health System, Miami, FL

Table S1. Baseline characteristics, clinical presentation and transcranial Doppler characteristics according to date of presentation.

	Date of presentation				
	January 2010 to December 2020 **n=83**		January 2021 to June 2022 **n=72**		*p value*
**Demographics**					
Age in years, median (SD)	70.1	(14.0)	66.1	(16.8)	0.11
Male, n (%)	52	(62.6)	33	(45.8)	0.04
Race/ethnicity, n (%)					<0.01
Non-Hispanic White	25	(30.1)	19	(26.4)	
Non-Hispanic Black	1	(1.2)	16	(22.2)	
Hispanic	44	(53.0)	36	(50.0)	
Other	13	(15.7)	1	(1.4)	
**Vascular risk factors**					
Hypertension, n (%)	63	(75.9)	54	(75.0)	0.90
Diabetes Mellitus, n (%)	25	(30.1)	27	(37.5)	0.33
Hyperlipidemia, n (%)	28	(33.7)	30	(41.7)	0.31
Current smoker, n (%)	27	(32.5)	8	(11.1)	<0.01
Previous stroke, n (%)	14	(16.9)	12	(16.7)	0.97
**Clinical presentation**					
Pre-morbid mRS, median (IQR)	0	(1)	0	(1)	0.72
NIHSS, median (IQR)	16	(11)	14	(8)	0.10
Vessel occluded, n (%)					0.20
ICA	11	(13.3)	16	(22.2)	
M1	50	(60.2)	34	(47.2)	
M2	22	(26.5)	22	(30.6)	
ASPECTS, media (IQR)	9	(2)	9	(2)	0.28
Stroke etiology, n (%)					0.35
Cardioembolic	32	(38.6)	33	(45.8)	
Large-artery disease	14	(16.9)	8	(11.1)	
Undetermined	34	(41.0)	25	(34.7)	
Other	3	(3.6)	6	(8.3)	
IV-tPA bridging therapy, n (%)	43	(51.8)	29	(40.3)	0.15
**Endovascular therapy**					
Time-to-puncture in minutes, median (IQR)	86.5	(53)	100	(63)	0.29
First-pass revascularization, n (%)	42	(51.2)	41	(57.8)	0.21
mTICI score, n (%)					0.25
2b	21	(25.3)	16	(22.2)	
2c	19	(22.9)	10	(13.9)	
3	43	(51.8)	46	(63.9)	
**Transcranial Doppler**					
Time to TCD in hours, median (IQR)	20.8	(15.4)	16.8	(16.2)	0.08
TIBI grades, n (%)					0.05
Grade ≤3	12	(14.5)	7	(9.7)	
Grade 4	24	(28.9)	11	(15.3)	
Grade 5	47	(56.6)	54	(75.0)	
MFV in cm/s, mean (SD)	65.6	(38.1)	56.2	(21.5)	0.06
PSV in cm/s, mean (SD)	111.1	(54.5)	98.9	(36.3)	0.11
PI, mean (SD)	1.2	(0.5)	1.2	(0.4)	0.47

Abbreviations ASPECTS Alberta Stroke Program Early CT Score; IQR interquartile range; IV-tPA intravenous tissue plasminogen activator; mTICI modified Thrombolysis in Cerebral Infarction; NIHSS National Institutes of Health Stroke Scale; TCD transcranial Doppler; SD standard deviation.

Table S2. Baseline characteristics, clinical presentation and transcranial Doppler characteristics according to early neurological improvement and symptomatic intracranial hemorrhage.

	Early neurological improvement			Symptomatic intracranial hemorrhage		
	Yes (n=66)	No (n=89)	*p*	Yes (n=16)	No (n=139)	*p*
**Demographics**						
Age in years, median (SD)	68.4 (12.7)	68.2 (17.3)	0.92	70.0 (18.7)	68.1 (15.1)	0.63
Female, n (%)	35 (53.0)	50 (56.2)	0.70	7 (43.8)	78 (56.1)	0.35
Race/ethnicity, n (%)						
Non-Hispanic White	22 (33.3)	22 (24.7)	0.18	2 (12.5)	42 (30.2)	0.40
Non-Hispanic Black	4 (6.1)	13 (14.6)		2 (12.5)	15 (10.8)	
Hispanic	32 (48.5)	48 (53.9)		11 (68.8)	69 (49.6)	
Other	8 (12.1)	6 (6.7)		1 (6.3)	13 (9.4)	
**Vascular risk factors**						
Hypertension, n (%)	52 (78.8)	65 (73.0)	0.41	12 (75.0)	105 (75.5)	1.00
Diabetes Mellitus, n (%)	24 (36.4)	28 (31.5)	0.52	8 (50.0)	44 (31.7)	0.14
Hyperlipidemia, n (%)	25 (37.9)	33 (37.1)	0.92	6 (37.50)	52 (37.4)	0.99
Current smoker, n (%)	17 (25.8)	18 (20.2)	0.42	3 (18.8)	32 (23.0)	1.00
Previous stroke, n (%)	14 (21.2)	12 (13.5)	0.20	1 (6.3)	25 (18.0)	0.31
**Clinical presentation**						
NIHSS, median (IQR)	14.5 (10)	15 (10)	0.87	17.5 (6.5)	15 (10)	0.21
Occluded vessel, n (%)						
ICA	12 (18.2)	15 (16.9)	0.42	3 (18.8)	24 (17.3)	0.72
M1	32 (48.5)	52 (58.4)		10 (62.5)	74 (53.2)	
M2	22 (33.3)	22 (24.7)		3 (18.8)	41 (29.5)	
ASPECTS, median (IQR)	9 (2)	9 (1)	0.04	8 (2.5)	9.0 (2)	0.04
Stroke etiology, n (%)						
Cardioembolic	27 (40.9)	38 (42.7)	0.40	10 (62.5)	55 (39.6)	0.33
Large-artery disease	8 (12.1)	14 (15.7)		1 (6.3)	21 (15.1)	
Undetermined	29 (43.9)	30 (33.7)		4 (25.0)	55 (39.6)	
Other	2 (3.0)	7 (7.9)		1 (6.3)	8 (5.8)	
**Endovascular Therapy**						
IV-tPA bridging therapy, n (%)	35 (53.0)	37 (41.6)	0.16	9 (56.3)	63 (45.3)	0.41
Time-to-puncture in minutes, median (IQR)	100.5 (68)	87 (47)	0.26	101.5 (31)	92 (59)	0.45
First-pass revascularization, n (%)	42 (65.6)	41 (46.1)	0.02	12 (75.0)	71 (51.8)	0.22
mTICI score, n (%)						
2b	16 (24.2)	21 (23.6)	0.95	2 (12.5)	35 (25.2)	0.54
2c	13 (19.7)	16 (18.0)		4 (25.0)	25 (18.0)	
3	37 (56.1)	52 (58.4)		10 (62.5)	79 (56.8)	
**Transcranial Doppler**						
TIBI grades, n (%)						
Grade ≤3	3 (4.6)	16 (18.0)	0.02	4 (25.0)	15 (10.8)	0.19
Grade 4	13 (19.7)	22 (24.7)		4 (25.0)	31 (22.3)	
Grade 5	50 (75.8)	51 (57.3)		8 (50.0)	93 (66.9)	
MFV in cm/s, mean (SD)	65.8 (34.4)	57.9 (29.3)	0.13	53.9 (29.3)	62.1 (32.0)	0.33
PSV in cm/s, mean (SD)	110.4 (47.7)	101.7 (46.7)	0.26	100.3 (49.6)	106.0 (47.0)	0.65
PI, mean (SD)	1.1 (0.4)	1.2 (0.5)	0.08	1.4 (0.8)	1.1 (0.4)	0.04

Abbreviations ASPECTS Alberta Stroke Program Early CT Score; IQR interquartile range; IV-tPA intravenous tissue plasminogen activator; MFV mean flow velocity; mRS modified Rankin Score; mTICI modified Thrombolysis in Cerebral Infarction ; NIHSS National Institutes of Health Stroke Scale; PI pulsatility index; PSV peak systolic velocity; SD standard deviation; TIBI Thrombolysis in Brain Ischemia.

Table S3. Association between in-hospital outcomes and Transcranial Doppler parameters s measured within 48 hours of endovascular therapy after adjusting for age at presentation, initial National Institutes of Health Stroke Scale (NIHSS) score and the Alberta Stroke Program Early CT Score (ASPECTS), modified Treatment in Cerebral Ischemia score, and site of vessel occlusion.

	**Discharge mRS 0-2** aOR (95% C.I.)^	**Favorable destination** aOR (95% C.I.)^	**In-hospital mortality** aOR (95% C.I.)^	**Early neurological improvement** aOR (95% C.I.)^	**Symptomatic ICH** aOR (95% C.I.)^
**TIBI grades**					
TIBI grade 4 vs. 5	0.68 (0.25-1.83)	0.92 (0.36-2.32)	0.88 (0.20-3.78)	0.62 (0.27-1.41)	1.27 (0.33-4.92)
TIBI grade ≤3 vs. 5	0.09 (0.01-0.79)*	0.24 (0.07-0.80)*	0.88 (0.22-2.37)	0.19 (0.05-0.71)*	3.69 (0.85-16.01)
**TCD parameters**					
MFV (cm/s)	1.01 (0.99-1.02)	1.01 (0.99-1.02)	1.00 (0.99-1.02)	1.01 (0.99-1.02)	0.99 (0.96-1.01)
PSV (cm/s)	1.00 (0.99-1.01)	1.00 (0.99-1.01)	1.00 (0.99-1.01)	1.00 (0.99-1.01)	0.99 (0.98-1.01)
PI	0.64 (0.21-1.98)	0.24 (0.09-0.67)*	1.99 (0.71-5.60)	0.45 (0.19-1.13)	2.02 (0.66-6.20)

^ Logistic regression model adjusted for age at presentation, initial National Institutes of Health Stroke Scale (NIHSS) score and the Alberta Stroke Program Early CT Score (ASPECTS), modified Treatment in Cerebral Ischemia score, and site of vessel occlusion; aOR for MFV, PSV and PI are per one unit increase.

* p value <0.05

Abbreviations ICH intracranial hemorrhage; MFV mean flow velocity; mRS modified Rankin Score; PI Pulsatility index; PSV peak systolic velocity; TCD transcranial Doppler; TIBI Thrombolysis in Brain Ischemia; aOR adjusted odds ratio
